# Harnessing technology and portability to conduct molecular epidemiology of endemic pathogens in resource-limited settings

**DOI:** 10.1093/trstmh/traa086

**Published:** 2020-09-18

**Authors:** Christina L Faust, Kirstyn Brunker, Diana Ajambo, Mary Ryan, Arinaitwe Moses, Candia Rowel, Robert Mandela Wangoola, Eddie M Wampande, Andrew Guma, Moses Adriko, Poppy H L Lamberton, Edridah M Tukahebwa, Albert Mugenyi, Charles Waiswa

**Affiliations:** Institute of Biodiversity, Animal Health and Comparative Medicine, University of Glasgow, Glasgow G12 8QQ, UK; Wellcome Centre for Integrative Parasitology, University of Glasgow, Glasgow G12 8QQ, UK; Institute of Biodiversity, Animal Health and Comparative Medicine, University of Glasgow, Glasgow G12 8QQ, UK; Vector Control Division, Ministry of Health, Kampala, Uganda; Glasgow Centre for International Development, University of Glasgow, Glasgow G12 8QQ, UK; Vector Control Division, Ministry of Health, Kampala, Uganda; Vector Control Division, Ministry of Health, Kampala, Uganda; Coordinating Office for the Control of Trypanosomiasis in Uganda, Kampala, Uganda; Department of Biotechnical and Diagnostic Sciences, Makerere University, Kampala, Uganda; Vector Control Division, Ministry of Health, Kampala, Uganda; Vector Control Division, Ministry of Health, Kampala, Uganda; Institute of Biodiversity, Animal Health and Comparative Medicine, University of Glasgow, Glasgow G12 8QQ, UK; Wellcome Centre for Integrative Parasitology, University of Glasgow, Glasgow G12 8QQ, UK; Vector Control Division, Ministry of Health, Kampala, Uganda; Coordinating Office for the Control of Trypanosomiasis in Uganda, Kampala, Uganda; Coordinating Office for the Control of Trypanosomiasis in Uganda, Kampala, Uganda

## Abstract

Improvements in genetic and genomic technology have enabled field-deployable molecular laboratories and these have been deployed in a variety of epidemics that capture headlines. In this editorial, we highlight the importance of building physical and personnel capacity in low and middle income countries to deploy these technologies to improve diagnostics, understand transmission dynamics and provide feedback to endemic communities on actionable timelines. We describe our experiences with molecular field research on schistosomiasis, trypanosomiasis and rabies and urge the wider tropical medicine community to embrace these methods and help build capacity to benefit communities affected by endemic infectious diseases.

Ebola, yellow fever, Zika and COVID-19 outbreaks in the last few years have benefitted from real-time sequencing of pathogens to understand the transmission and evolution of these emerging viruses.^1^ The portability and low cost of MinION sequencers have captured the imagination of researchers and the public, but also made ‘lab in a suitcase’ sequencing a possibility. Studying genetics no longer requires a laboratory facility with bright lights and hefty equipment: the technology and creative solutions facilitating PCR product-free and sterile environments nearly anywhere have enabled a rapid response to emerging pathogens. Although these epidemics dominate headlines, we argue that these opportunities should also be extended to improve diagnostics, understanding of transmission dynamics and to design more effective intervention strategies to target endemic infectious pathogens in low and middle income countries (LMICs). Moreover, building core capacity and routine molecular surveillance in the areas most burdened by infectious disease is critical to ensure epidemic preparedness.

There is immense potential to use new technologies and lowered equipment costs to move capacity into those regions that are most impacted by infectious diseases. Countless molecular work currently relies on shipping samples to complete processing, often with long delays and removed from those who collect samples and deal with pathogens on a daily basis. The declining cost of portable PCR machines, quantitative PCR and sequencers make them a more feasible option and several are robust to field conditions. These portable pieces of equipment mean that machines can stay in country and be moved on site to study specific endemic diseases in real time. They can even be used in stationary laboratories to build scalable molecular capacity at reference laboratories. Consumables are a real challenge, as we discuss below, but new protocols, tools and reagents are lowering hurdles for conducting molecular work.

A key bottleneck to developing molecular capacity in the field, or in areas with intermittent supply, is access to reliable electricity. Power is required to run machines, heat samples during lysis and to maintain a cold chain for essential reagents and unstable nucleic acids. Several creative solutions have removed the need for electricity, ranging from hand-powered centrifuges to temperature-stable polymerases. A range of commercially available reagents now no longer need refrigeration, require a ‘hot-start’ to enable PCR set-up at room temperature, or both. These advances reduce cross-amplification and enable accurate molecular work to be carried out in the field. In our work diagnosing zoonotic human African trypanosomiasis (rHAT; causative agent *Trypanosoma brucei rhodesiense*), we found that results obtained in the field with a modified extraction protocol and temperature-stable reagents were consistent with results later obtained with the same samples in a molecular lab at Makerere University (Figure 1A). If cold chains are necessary, liquid nitrogen, dry ice and reusable ice packs can work for short-term options (days to weeks). When we could not avoid power altogether, we used solar panels and lithium batteries to run low wattage centrifuges and PCR machines. Combining resources with the Coordinating Office for the Control of Trypanosomiasis in Uganda and the Vector Control Division, Ministry of Health, Uganda, allowed purchase of the equipment and enabled a field-deployable molecular lab.

**Figure 1.**
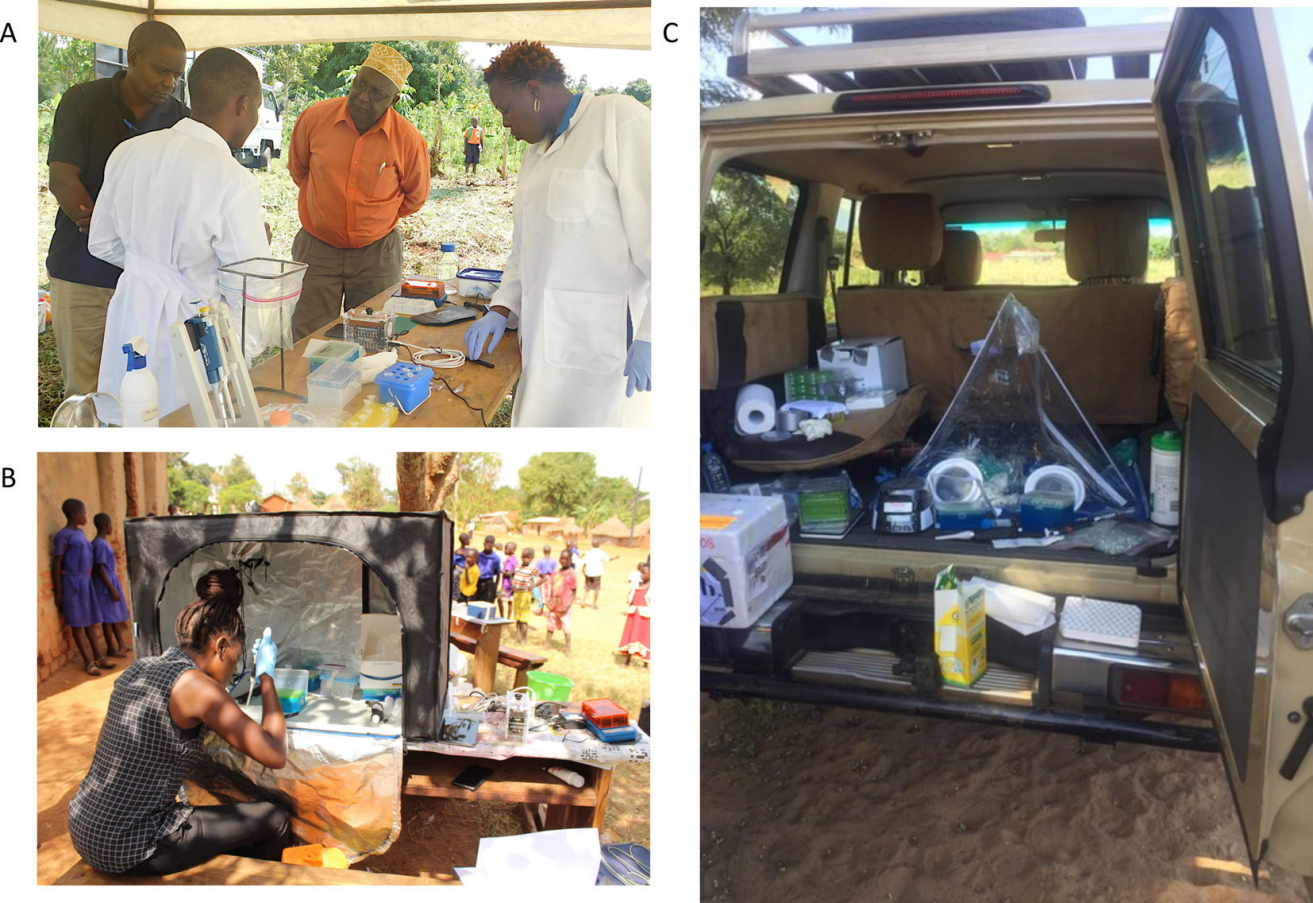
Field deployment of molecular laboratories in Uganda and Tanzania (A) for diagnosing *Trypanosoma brucei rhodesiense* (a causative agent of human African trypanosomiasis), (B) zoonotic schistosomes and (C) rabies genomic surveillance.

The constraints of nucleic acid extraction can also be a major hindrance to molecular work. New methods allow DNA extraction from plants, animals and environmental samples at room temperature, or nearly room temperature. We have been using FTA^TM^ cards to lyse cells, inactivate pathogens and bind DNA to the matrix in field conditions. After 1 h of drying, DNA can be eluted from tissue samples and whole organisms (i.e. parasites) using modified solutions then used directly in PCR reactions (Figure 1B). Expensive commercially available kits can rapidly extract DNA within minutes in the field and possess single-use contained columns to prevent cross-contamination. If more extensive lysis steps are required before sample extraction, there are portable tissue homogenisers and water baths that can be powered by low wattage. In a pinch, toothpicks, thermometers, candles and charcoal stoves have all been used successfully as substitutes.

An additional challenge of conducting molecular work in the field is maintaining a safe working space. The ARTIC network (https://artic.network/ebov) has developed accessible protocols to inactivate, amplify and sequence viral samples in the field in a robust, sensitive and safe manner. The development of portable laboratories has inspired many of the kit and processing pipelines described here. These researchers have used portable glove boxes to help contain infectious material before they are inactivated, and made their packing lists and operating protocols open for others to learn from. Portable glove boxes help maintain safety, keep the space sterile, and can be lightweight and packable (Figure 1C). Portable UV lights and bleach are both cheap and effective in sterilising and preventing cross-contamination of workspaces. However, the scope of work will be constrained by the level of containment required for specific pathogens.

Field laboratories are increasingly being used for in-depth studies that go beyond diagnosis, even generating whole genomes. Our published and validated pipeline for rabies genomic research can be applied in low-resource settings and will be an important resource for informing elimination campaigns.^2^ The Cassava Virus Action Project has developed genomic pipelines to improve food security in Africa, using whole genome sequencing to characterise the diversity of cassava viruses infecting a farmer's field and provide recommendations of which strains of cassava would be ideal for replanting.^3^ This provides increases in yield for the individual farmer but also provides important information on how these cassava viruses are evolving in response to human control interventions.

The application of these new technologies must also be met with a commitment to training local researchers. Funding for some of our training work has come through the Global Challenges Research Fund, part of the UK's Official Development Assistance commitment. Funding initiatives that support exchange and training opportunities are crucial for developing capacity and we hope that such important financial support will continue. In general, LMICs suffer from a lack of bioinformatic expertise, but dedicated new initiatives such as the Pan African Bioinformatics Network show promising growth in this area.^4^

One of the most beneficial aspects of field laboratories is the speed at which samples can be collected and analysed. These fast pipelines are revolutionising how we study outbreaks and control important animal, plant and human diseases. Deployable rabies virus sequencing has lowered costs and reduced sample-to-sequence lag times from up to 6 months (with sample export) to a matter of days, providing rapid, actionable epidemiological insights for rabies control efforts.^2^ The nearly instantaneous feedback to affected communities can help avoid fatigue that communities experience when they must wait months or even years for results. Whole genome sequencing of bacteria in the Philippines is informing antimicrobial resistance management and policy in real time.^5^ Field molecular laboratories can help identify infected animals using pen-side tests and make informed decisions on culling and treatment to limit future human cases and economic losses.^6^

Despite these obvious benefits, we believe one of the biggest challenges in conducting molecular work is sourcing and paying for consumables. Local suppliers in LMICs offer a limited range of molecular reagents, items are often out of stock or take months to deliver and are prohibitively expensive. Importation regulations exacerbate the delays and expense of acquiring necessary reagents, and customs delays compromise the integrity of reagents. In our experience, airway bills, including duty tax and in-transit cold storage costs, often exceed the price of the reagents themselves. A secure, affordable supply chain will ultimately ensure the sustainability of molecular research in LMICs. While global companies continue to monopolise prices this will remain a major challenge to research implementation in these areas. Companies simply must do more to improve supply chains and the accessibility of their products. The wider research community is also beginning to develop low-cost ‘do-it-yourself’ alternatives for some reagents, which provide alternatives to some of these consumable needs (i.e. https://bomb.bio/about/).

By bringing the lab to the sample, it is possible to avoid delays caused by shipping samples and long lag times in communicating results back to the affected communities. It is essential to build physical and human capital to carry out this work within endemic settings and ensure that consumables are not a limiting factor in conducting this work. Helicopter science limits the perspective of an endemic disease, and building confidence in the application of molecular methods throughout academic and government human and animal health institutions will only benefit from these advances.
